# Perspectives of personalized immunomodulation in dentistry

**DOI:** 10.1186/1878-5085-5-S1-A121

**Published:** 2014-02-11

**Authors:** Alexei Gratchev, Julia Kzhyshkowska

**Affiliations:** 1 Medical Faculty Mannheim, Ruprecht-Karls University of Heidelberg, Theodor-Kutzer Ufer 1-3, 68167, Mannheim, Germany

## 

Inflammation is a normal reaction of an organism on infection or injury. Being a multistep and tightly regulated process, inflammation involves various cells of innate and adaptive immune systems. Macrophages are the key cells of innate immunity responsible for initiation, control and downregulation of inflammation. Malfunction of macrophages is responsible for the development of periodontitis and their exaggerated reaction plays an important role in the onset and severity of the peri-implant diseases. Activation of macrophages may be achieved by microbial products or inflammatory cytokines including IFNgamma thereby leading to the development of type 1 phenotype (M1). On the other hand, anti-inflammatory cytokines or Th2-cells-associated cytokines such as IL-4 induce type 2 macrophages (M2). While M1 are responsible for inflammation initiation and amplification, M2 are needed to reduce inflammatory activity and to initiate tissue repair and healing. The balance between type 1 and type 2 activation has to be strongly maintained to prevent development of chronic inflammation or fibrosis. It is in the nature of human population heterogeneity that in immune system of some patients type 1 or type 2 reaction prevails. This individual property has a genetic background and may lead to unexpected complications of the treatment. Our research on macrophage biology enabled us to develop a cell-based system that can be used both as a diagnostic tool and as a model for development of novel immunomodulatory therapies capable of reducing complication during treatment. This cell-based system may be used for prediction of unwanted reaction of the patient to the implant material, identification of undesired immune status of the organism and to design personalized immunomodulatory strategy to improve the outcome of the treatment. Innovative approaches for modulation of macrophage reaction to dental implants are developed in our laboratory.

